# Block V RTX Domain of Adenylate Cyclase from *Bordetella pertussis*: A Conformationally Dynamic Scaffold for Protein Engineering Applications

**DOI:** 10.3390/toxins9090289

**Published:** 2017-09-17

**Authors:** Beyza Bulutoglu, Scott Banta

**Affiliations:** Department of Chemical Engineering, Columbia University, 500 W 120th Street, New York, NY 10027, USA

**Keywords:** protein engineering, RTX domain, β-roll domain, hydrogels, bioseparations, biomolecular recognition

## Abstract

The isolated Block V repeats-in-toxin (RTX) peptide domain of adenylate cyclase (CyaA) from *Bordetella pertussis* reversibly folds into a β-roll secondary structure upon calcium binding. In this review, we discuss how the conformationally dynamic nature of the peptide is being engineered and employed as a switching mechanism to mediate different protein functions and protein-protein interactions. The peptide has been used as a scaffold for diverse applications including: a precipitation tag for bioseparations, a cross-linking domain for protein hydrogel formation and as an alternative scaffold for biomolecular recognition applications. Proteins and peptides such as the RTX domains that exhibit natural stimulus-responsive behavior are valuable building blocks for emerging synthetic biology applications.

## 1. Introduction

*Bordetella pertussis* is a Gram-negative bacteria, first isolated in 1906 and is the causative agent of whooping cough [1]. The adenylate cyclase (CyaA) toxin is one of the major virulence factors of this organism [2,3]. CyaA belongs to the repeat-in-toxin (RTX) family of proteins and in its active form, this 1706 amino acid enzyme is able to invade eukaryotic cells. It is composed of two domains: an N-terminal cell-invasive catalytic domain composed of 400 amino acids and a C-terminal 1306 amino acid domain composed of five consecutive RTX domains [4,5,6]. The catalytic domain of the enzyme is activated upon binding of eukaryotic calmodulin [7]. Upon activation, it upregulates cAMP production, interfering with cellular signaling pathways. The hemolytic RTX domain is responsible for cell binding and for the translocation of the catalytic domain across the cell membrane of the target cells. Upon secretion, CyaA binds to the surface receptor CD11b/CD18 displayed on immune cells such as neutrophils and dendritic cells [6,8,9,10]. A recent immunization study showed that the RTX domain by itself constitutes a promising alternative to using the entire CyaA protein in vaccine development [11]. 

The RTX domain of CyaA has a highly repetitive sequence composed of 41 glycine and aspartic acid-rich repeat nonamer sequences [12]. RTX domains bind calcium and undergo a structural transition, which facilitates the secretion of the attached catalytic domain via the bacterial type I secretion system. It has been shown that the Block V RTX domain and the C-terminal capping group (residues 1529–1681) are required for the proper activity of the CyaA toxin [13,14,15,16]. Structural models have been proposed for the unstructured, calcium-free RTX domain of CyaA, as well as for the calcium-bound folded state, which forms the β-roll secondary structure [17]. The crystal structure of this domain was recently reported, and NMR experiments have helped confirm the folding mechanism of the RTX domain. Folding is directional, with calcium binding starting at the C-terminus of the β-roll and progressing towards the N-terminus [18]. Another recent study reported a comprehensive 3D structural characterization of the calcium-bound folded form of CyaA. The active toxin was stable at room temperature for several days and the presence of calcium ions enhanced its thermal stability [19]. 

We have been exploring the Block V RTX domain for synthetic biology applications and have contributed to the characterization of this domain as a part of these activities. In this review, we will first present the advantages of repeat proteins as protein engineering scaffolds. Then, we will summarize some of the characterization work that has been done with the Block V RTX domain using protein engineering approaches. Lastly, we will discuss several applications where this peptide has been engineered for new calcium-mediated functions, including as a precipitation tag for bioseparations, a cross-linking domain for protein hydrogel formation and as a structural switch for controllable biomolecular recognition. 

## 2. Repeat Proteins as Scaffolds for Protein Engineering

Proteins with repetitive structural architectures have gained attention as versatile scaffolds for protein engineering. They have repetitive sequences where conserved residues in the repeats often form the structural framework of the protein as well as the hydrophobic protein core. The variable residues in the repeats are often solvent-accessible and can form interactions with other molecules. One advantage of these repeat systems is that proteins can be rationally engineered by mutating, adding, or deleting individual repeats, and changing the number of repeats can often be accommodated without impairing the global folding architecture of the protein [20,21,22,23,24]. In order to place the use of the Block V RTX domain of CyaA into perspective for protein engineering applications, we first describe some related examples of repeat protein scaffolds that have been extensively engineered for various applications. 

### 2.1. Designed Ankyrin Repeat Proteins (DARPins)

Ankyrin repeat proteins are comprised of repeating units of 33 amino acids, each of which folds into a β-turn followed by two antiparallel α-helices. In a single ankyrin repeat protein, up to 29 repeats can be present [25,26,27,28]. It was shown that a C-terminal capping group is essential for proper folding of these proteins. Both natural and designed synthetic protein libraries (DARPins) have been created and selection strategies have been used (phage display, ribosome display, cell-surface display) to identify DARPins with unique molecular recognition capabilities [29]. Working library sizes of up to 10^12^ were reported for selections via ribosome display [30,31]. In these studies, seven positions were randomized per designed repeat module at positions with potential target interaction (i.e., β-turns and the first α-helices). DARPins have been engineered for applications including biosensors, tumor targeting and drug delivery; and in a clinical application for the prevention of macular degeneration [32,33,34,35].

### 2.2. Leucine Rich Repeats (LRRs)

LRRs are among most-studied repeat domains. These repeats can have up to 29 residues, 11 of which constitute a conserved consensus sequence. The secondary structure of the repeats is dominated by α-helices [36,37]. Primarily present in eukaryotic proteins, these domains are responsible for various protein-protein interactions [38]. Interestingly, some jawless vertebrates employ variable lymphocyte receptors (VLRs) composed of these repeat domains in their adaptive immune system [39,40]. The potential diversity of VLRs was explored in experiments with lampreys. Following anthrax immunization, this jawless fish was capable of generating a large amount of anthrax-specific VLRs, similar to the mammalian immune response [41]. Leucine-rich repeat motifs are also found to serve similar roles in insects and plants [42,43]. LRRs were proposed to serve as artificial receptors and minimized, synthetic LRR domains, composed of three repeat sequences, were shown to fold into similar structures compared to native variants [44]. These repeat domains were also utilized as alternative binding-scaffolds, designed and selected against various targets including hen egg lysozyme [45]. In another study, LRRs assembling into structures with predefined geometries, e.g., ring-shaped, were designed via a structure-based computational approach [46].

### 2.3. Other Repeat Proteins

There are many other repeat proteins, which play a role in ligand binding and the mediation of protein complex interactions [22]. Armadillo repeats are composed of 42 amino acids and up to 15 repeats form a secondary structural motif of three α-helices [47]. Pentatrico peptide repeat (PPR), tetratrico peptide repeat (TPR) and HEAT repeat domains are also α-helical [48,49]. TPRs have a repeat size of 34 amino acids and most commonly, three repeats are stacked together. HEAT motifs have repetitive units of 37–47 residues and their number of repeats range between three and 36. A recent study introduced a general computational method for designing new repeat proteins with different sets of sequences, which were applied to ankyrin, armadillo, TPR, HEAT, and LRRs [50]. These scaffolds have been used for different applications including in vivo manipulation of gene expression and as small molecule and peptide binders [51,52].

## 3. Block V RTX Domain of Adenylate Cyclase from *Bordatella pertussis*

A central feature of the repeat scaffolds described above is structural stability. As these proteins are engineered for molecular recognition capabilities (often with therapeutic applications), it is critical that the structures are robust and stable. β-roll forming domains, such as the Block V RTX domain from CyaA, are also repeat protein scaffolds, however, the β-roll repeat structure only forms in the presence of calcium. Thus, the calcium responsiveness provides a means to reversibly transition the peptides from an unfolded state to a folded state. Therefore, RTX domains could serve as unique and versatile protein scaffolds, and the Block V RTX domain offers a unique combination of favorable features: its calcium responsiveness enables a control mechanism over its function, and its repetitive sequence allows for the engineering of the peptide without the disruption of the switching mechanism. In addition, the two β-sheet faces in the folded state can be explored as regions capable of interacting with other peptides, proteins and small molecules. These features enable the Block V RTX domain to present new opportunities in protein engineering and synthetic biology [53].

In addition to CyaA, RTX domains are present in lipases (e.g., lipase A from *Serratia marcescens*) and proteases (e.g., alkaline protease from *Pseudomonas aeruginosa*), in Gram-negative bacteria [54,55,56]. The RTX domains are composed of repeats of GGXGXDXUX, where U represents an aliphatic amino acid and any residue can be found in position X. The calcium ions are bound by highly conserved aspartic acids so that the ions are located between two consecutive turns of the β-roll structure. The RTX proteins become biologically activated upon calcium binding, allowing them to serve as molecular chaperones or as the folding nuclei for the rest of the protein. Virulence factors, such as *Escherichia coli* α-hemolysin (HlyA), depend on their RTX domains for the initial interactions with the target cells [6,9,55,57,58]. Thus, nature employs these domains as a calcium-dependent switching mechanism that can be used to localize protein activity to calcium-rich environments.

**Figure 1:**
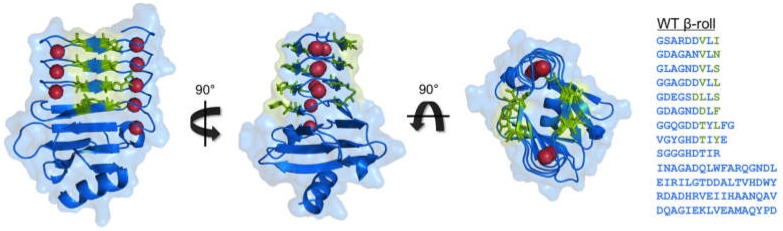
The structure of the Block V RTX domain (β-roll) and its protein sequence. The bound calcium ions are shown in red. Positions where site-directed mutagenesis has been performed are highlighted in green (eight residues on each face of the peptide [66,67,68,69] and Asp1570 [70]). All figures were rendered in PyMOL using PDB file 5CVW.

The Block V domain of the CyaA protein is one of the most studied RTX sequences and is the basis of the majority of protein engineering efforts in this area. The RTX domains are disordered in the intracellular space, where the calcium concentration is low [59,60]. Upon secretion into the extracellular space, where the calcium concentrations are higher, the domains fold into the β-roll secondary structure, a β-helix composed of turns and parallel β-sheets as shown in Figure 1 [16,18]. Several groups have characterized the Block V β-roll peptide using different biophysical techniques. It has been established that there is cooperativity in the conformational change of the β-roll as the binding of one calcium ion facilitates the formation of the binding site for the next ion. Thus, the binding of each calcium ion leads to an increase in the affinity of the adjacent binding site, producing a polarized folding mechanism that propagates from the C-terminus towards the N-terminus. In addition, a C-terminal capping group was found to be necessary for proper folding and function [13,18,61,62,63,64,65].

**Figure 2:**
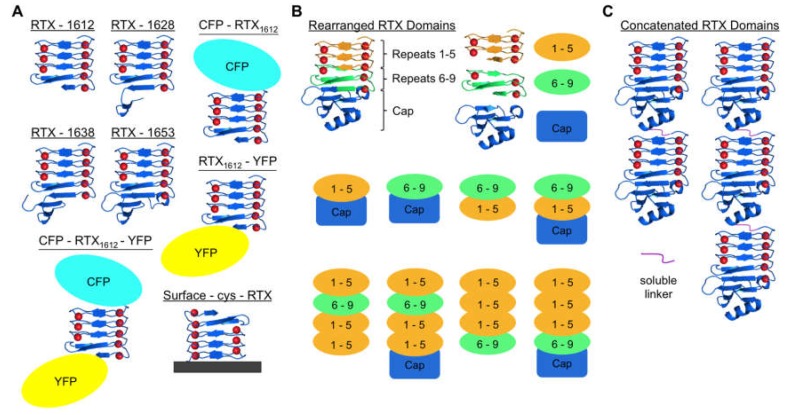
Schematic diagrams of examples of engineered Block V RTX domain variations. (**A**) Truncated peptides were constructed to examine the minimal natural C-terminal flanking sequence needed for calcium responsiveness [61]. Cyan fluorescence protein (CYP) and yellow fluorescence protein (YFP) were used to characterize the conformational change of the domain by FRET, and this led to the discovery that YFP could serve as a C-terminal capping group [70]. Tethering of the RTX domain to a QCM crystal allowed the surface to function as a terminal capping group [71]; (**B**) rearranged RTX domains were created to explore sequence modularity and functionality. Rearranged RTX repeats were built with or without the capping group [64]; and (**C**) different numbers of full-length RTX domains (each with C-terminal caps) were concatenated with linkers and used in biomolecular recognition and protein hydrogel studies [68,69].

Circular dichroism (CD) spectroscopy and bis-ANS dye binding have been used to characterize the folding of the Block V domain, however, these techniques require large amounts of purified protein, and are not high-throughput techniques. Thus, we investigated the folding mechanism of the β-roll peptide via Förster resonance energy transfer (FRET) of genetically appended fluorescent proteins [70] (Figure 2A). This allowed for the calcium-induced folding mechanism to be evaluated in a larger experimental space so that the impact of different ions, and ionic strengths could be rapidly evaluated, even in crude protein preparations. This also allowed for the rapid evaluation of the impact of site-directed mutations on the calcium-induced folding of the RTX domain. 

Interestingly, the above-mentioned FRET studies led to the discovery that when a fluorescent protein is added to the Block V sequence in place of the C-terminal capping group, the chimeric protein still enabled calcium-induced folding into the β-roll structure. This was confirmed with the addition of maltose binding protein (MBP) to the C-terminus of the peptide. It was shown that the folding of the Block V sequence requires some protein mass at the C-terminus. The native capping group enabled folding at the lowest calcium concentration, but folding could occur with a fluorescent protein or MBP at the C-terminus at higher calcium concentrations. Thus, it was concluded that the capping group provides entropic stabilization of the C-terminus which is necessary for the folding of the RTX domain into the β-roll structure [61]. Schematic representations of some of the different versions of the RTX domain peptides constructed for these investigations are shown in Figure 2A.

The results were expanded and confirmed using quartz crystal microbalance (QCM)-based studies. Cysteine amino acids were added to the ends of the capped and un-capped Block V peptides and they were used to immobilize the peptides in different orientations onto the gold surface. The folding of the RTX domains could then be monitored by the change in the resonance frequency of the peptides as the β-roll formation affected the local mass and viscosity near the crystal surface. It was found that the quartz crystal could also serve as a capping group, stabilizing the C-terminus of the peptide and allowing for calcium-dependent folding (Figure 2A) [71]. Interestingly, some calcium-dependent changes were also seen when the peptides were immobilized by the N-terminus (and the C-terminus was uncapped) and it was assumed this was due to a molecular crowding effect on the surface. Overall, these results demonstrated that the Block V RTX peptide could be functionally immobilized onto a surface, and that the surface could be used to entropically stabilize the C-terminus.

## 4. Native RTX Domain Insertions for Introducing Calcium-Mediated Function

In addition to characterization studies, other interesting work focused on the design of new synthetic RTX domains and the use of these domains in different applications. The structural dynamics of native RTX domains have been used as tools for protein engineering. By inserting RTX domains into other proteins, calcium-dependent control can be incorporated into new systems. The first example of this approach involved the insertion of an RTX domain from *Serratia marcescens* serralysin into a 2-D protein mesh network. The RTX domain served as a spacer between two other proteins (6-phospho-β-galactosidase (PGAL)) and the spacing of the proteins in the mesh could be controlled by varying calcium concentrations [72]. This study demonstrated the use of a minimized RTX domain, composed of five repeats, as a calcium-dependent molecular switch. Chelation of the calcium ions resulted in the extended conformation of the domain, where the two PGAL molecules became more distant.

More recently, we have extended this approach by inserting the Block V RTX domain from CyaA into an active site loop of an enzyme. Alcohol dehydrogenase D (AdhD) from *Pyrococcus furiosus* prefers NADH over NADPH in the reductive direction and exhibits activity with a broad range of substrates, but is most active with secondary alcohols [73,74]. A loop in the substrate binding pocket of the enzyme was shown to be capable of affecting cofactor selectivity [75] and insertion of the Block V RTX domain into this loop led to the creation of an enzyme such that calcium impacted its NAD^+^-dependent activity with less of an effect on NADP^+^-dependent activity. Thus the insertion of the RTX domain into AdhD introduced a calcium-dependent “rheostat-like” switch that can be used to tune the cofactor preference of the enzyme [76].

## 5. Exploring the Order of RTX Domain Repeat Sequence Lead to Useful Precipitation for Bioseparations

As RTX domains are explored for synthetic biology applications, it is logical to create synthetic peptides with designed sequences that can be rationally used to extend or shorten the RTX domains and create useful modules for further protein engineering (Table 1). In one study, a synthetic RTX domain with eight identical repeats of GGSGNDNLS was constructed, which possessed a sub-optimal consensus sequence (e.g., the asparagine at position 7, which does not generally occur naturally). This synthetic peptide was shown to behave in a similar manner as the natural RTX domains [77]. In other work, shorter versions of synthetic RTX peptides composed of three and five β-strands were designed to serve as protein engineering scaffolds. However, the authors discovered that these peptides underwent reversible, metal-ion dependent polymerization, generating long protein filaments [78]. We explored RTX domains where a consensus RTX sequence (GGAGNDTLY) was concatenated together. However, peptides made from this sequence were not found to behave like wild-type Block V sequence, and precipitation was frequently observed instead of β-roll formation [65].

These results motivated the investigation of the length and ordering of the RTX repeats in the Block V peptide (Figure 2B). First, proteins were made where the native order of the RTX domains was retained, but the N-terminal repeats were either deleted or duplicated to extend the β-roll structure. These results were compared to molecules of the same length, but the ordering was altered so that C-terminal repeats were swapped with N-terminal repeats. For the peptides with native ordering, the extension or truncation of the sequence resulted in peptides capable of folding but higher concentrations of calcium were required as compared to the wild-type sequence. For the rearranged sequences, the calcium requirements for structural changes were dramatically higher, and a trend was observed where shorter sequences required more calcium than the longer sequences. Overall these results demonstrated the importance of the polarized folding mechanism and are consistent with the model where some sequences have higher affinities for calcium than others, and the higher affinity repeats are localized closer to the C-terminal capping group to enable cooperative folding from the C-terminal end [64]. 

Based on these results, we hypothesized that the precipitation behavior of the peptides with the consensus sequence could be enhanced by further extending the repeats of this sequence (Table 1). Peptides with 5, 9, 13, and 17 repeats of the sequence were created, and it was found that the longer peptides (13 and 17) led to the highest propensity to reversibly precipitate upon calcium addition. There has been an interest in fusing recombinant proteins of interest to stimulus-responsive tags that can reversibly precipitate upon changes in the temperature, pH or ionic condition of the buffer [79,80]. Therefore, we developed the 17-repeat peptide as a beta roll tag (BRT) for non-chromatographic protein purification [65]. This BRT was fused to different proteins, including green fluorescent protein (GFP) and the alcohol dehydrogenase (AdhD). Pure and active recombinant proteins were obtained via calcium-induced precipitation of the proteins from the *E. coli* cell lysate, followed by the protease cleavage of the BRT17 tag [65]. This platform was used by an undergraduate iGEM team for the purification of a tagged *Eco*RI restriction enzyme, where RTX-tagged enzyme retained its endonuclease activity after purification via calcium-mediated precipitation [81].

## 6. Engineered β-Roll Domains with Hydrophobic Faces for Self-Assembly and Protein Hydrogel Formation

In the folded state, the β-roll structure has two parallel β-sheet faces and each β-strand in the β-roll face has two residues with solvent-exposed side chains (Figure 1). These residues do not contribute to the stabilization of the protein, or to calcium binding, and they are highly variable among other RTX domains [16,63,70]. This creates an opportunity to vary these pairs of amino acids for desired new functionalities, without compromising the unique and specific environmentally-responsive behavior of this peptide.

Leucine zipper domains are alpha-helical, leucine-rich repeat proteins that are capable of forming dimers and tetramers via burying their hydrophobic regions in coiled-coil structures [82]. This intrinsic assembly behavior has been exploited for the development of protein biomaterials [83]. The helical domains have been fused to soluble peptides to create polymeric hydrogels where the cross-linking is mediated by the leucine zipper interactions [84,85]. We have appended these leucine zipper domains to various enzymes for the creation of enzymatically active hydrogels for different applications [86,87,88]. The assembly of these materials can be controlled through the introduction of conditions that disrupt the helical domain formation, such as changes in pH, temperature, or ionic strength [83,89,90]. 

**Figure 3:**
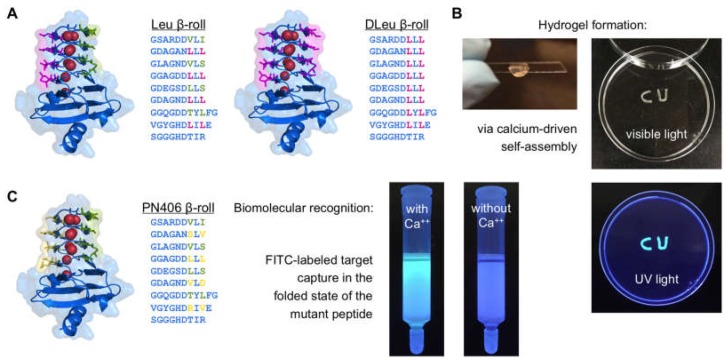
Engineered RTX domains for biotechnology applications. (**A**) Hydrogel-forming RTX peptides with their mutations highlighted in magenta. Bound calcium ions are shown in red. Leu β-roll has leucine residues at eight positions on one peptide face whereas DLeu β-roll has leucine residues on both faces; (**B**) images of proteinaceous hydrogels formed by these mutant constructs with hydrophobic faces; (**C**) PN406 mutant selected against lysozyme target via ribosome display. The randomized positions for library construction are shown in yellow in the primary sequence. The concatemer of this mutant was used for affinity chromatography applications where it captured the target molecule (lysozyme) in the presence of calcium, as shown in the picture on the right. This mutant was fused to the DLeu β-roll. The hydrogel formed by this construct retained the FITC-labeled target lysozyme. Leu, DLeu, and PN406 β-rolls were rendered in PyMOL using PDB file 5CVW.

Inspired by the leucine zipper domains, we mutated the eight residues on one face of the Block V β-roll peptide to leucine side chains [66]. This mutant peptide, along with its primary sequence is shown in Figure 3A. This resulted in the formation of a hydrophobic face upon addition of calcium, and this face was amenable for cross-linking like the leucine zipper domains. This mutant, Leu β-roll, was fused to a soluble linker domain and a leucine zipper domain. The leucine zippers self-assemble, independent of the calcium concentration. However, the β-roll domains remain disordered in the absence of calcium ions, preventing network assembly, and a viscous liquid was formed. Upon calcium addition, the β-roll domains fold, forming the leucine rich face, and this resulted in dimerization and network formation. Microrheology experiments were used to quantify the physical properties of the hydrogel and we found that elasticity of the hydrogel depended on the calcium concentration. Thus, the mutant RTX domain enabled the formation of a new class of “smart biomaterials” where hydrogel formation only occurs upon the addition of calcium [66].

We expanded on these results by exploring mutations on the other side of the symmetrical β-roll structure [67]. The amino acid residues located on both the front and back β-roll faces were mutated to leucine residues (named the DLeu β-roll, Figure 3A). This resulted in two hydrophobic interfaces that will form only in the presence of calcium. Calcium-dependent gelation was again observed when the mutant was attached to a soluble peptide and leucine zipper, and the introduction of the additional hydrophobic interface lowered the concentration of protein necessary for hydrogel formation. The improved cross-linking ability of DLeu β-roll mutant enabled the exploration of the RTX peptide as a stand-alone cross-linking domain. Proteins were made where the leucine zipper domains were eliminated and calcium-dependent gelation was demonstrated in a construct composed of the concatemer of the DLeu β-roll peptide and a globular protein (MBP) [67].

In a more recent work, this domain was fused to another mutant β-roll, which has been engineered to be capable of lysozyme binding (described below). The hydrophobic interfaces of the crosslinking DLeu β-roll domain actuated the self-assembly whereas the lysozyme binding β-roll domain captured the lysozyme within the assembled hydrogel network. By engineering the same residues on the β-roll scaffold for different purposes, distinct protein interfaces were constructed and combined. This work resulted in a biomaterial with dual functionality where both the assembly and the target capture functions can be controlled via changes in the calcium concentration (Figure 3B) [69]. 

## 7. Evolution of β-Roll Domains Exhibiting Calcium-Dependent Biomolecular Recognition

The amino acids on the faces of the Block V RTX domain can be mutated without affecting the calcium-dependent β-roll structure formation. Thus the RTX domain offers a unique capability among repeat proteins in that a binding face can be engineered while leaving the amino acids involved in the calcium-dependent conformational change unchanged. We explored whether the techniques used to engineer other repeat proteins, like the DARPins [91], could be used to introduce biomolecular recognition into the RTX domain [21,29]. 

RTX peptide libraries were created where the eight amino acids on one face of the peptide were randomized to express all 20 possible amino acids [68]. This library of mutant RTX domains was selected against immobilized lysozyme, serving as a model target protein, using ribosome display. Several mutants were identified with calcium-dependent affinities towards the target molecule including the PN406 mutant (Figure 3C). Further protein engineering tools were applied where the second face of the peptide as well as concatemers of the promising mutants (Figure 2C) were created and explored. The best mutant (PN406-PN406) was a concatamer of the selected PN406 peptide. The new protein bound lysozyme with a dissociation constant of 65 ± 28 nM but only in the presence of calcium. This mutant was used in affinity chromatography experiments to demonstrate the calcium-dependent control over this binding event. Target protein capture and release were demonstrated by simply changing the calcium concentration in the chromatography column. The mutant immobilized on the chromatography column was capable of forming interactions with the lysozyme in the column, in the presence of calcium. Target elution was induced upon removal of calcium ions, thus disabling the β-roll structure and reducing the protein interactions. The specificity of this new protein interaction was confirmed by repeating the same affinity experiments in the presence of non-specific *E. coli* proteins [68].

## 8. Summary and Conclusions

Nature has evolved the RTX domains as necessary participants in the type 1 secretion system, which enables protein structure and function to be directed extracellularly. Characterization of these novel domains can shed light on disease pathology, as well as to create new therapeutic opportunities. Protein engineering approaches have been used to elucidate the biophysical behavior of these domains providing new insights into the stability and folding mechanisms of these proteins. 

Since the RTX domains exhibit highly specific stimulus-responsive switching behavior leading to a well-characterized disordered-to-ordered structural transition, several groups have explored these domains as building blocks for synthetic biology applications. Native RTX domains have been used to control spacing between proteins, as well as to subtly alter the conformation of an enzyme. Designed RTX repeats have been utilized to construct new peptide materials, and to create a desirable phase change that can be exploited for bioseparations. Rationally-mutated β-roll domains have been created with hydrophobic faces that can be used to drive self-assembly leading to hydrogel formation. Most recently, directed evolution approaches have been used to select β-roll domains with affinity to a target protein (lysozyme) leading to a novel stimulus-responsive capture and release capability that has been demonstrated through protein affinity chromatography. 

It is becoming clear that conformationally-dynamic and/or intrinsically-disordered peptide motifs are playing important roles in biological processes and as these peptides are further explored and characterized, they will lead to further biophysical insights as well as additional biotechnological uses. In summary, the Block V RTX domain of CyaA, with its remarkable conformational behavior, demonstrates how fundamental insights into biomolecular mechanisms underpinning disease pathology can inspire new technologies. It is likely that further advances will soon be made in applications including nanomedicine, theranostics, artificial viruses, and other biotechnological areas and these efforts can be supported by fundamental studies in toxinology.

## Figures and Tables

**Table 1:** Synthetic RTX peptides with their sequences and number of repeats.

Sequence	No. of Repeats	Reference
GGSGNDNLS	8	[77]
GGSGSDLLK GNDVANWLK GGAGNDILE GGLGADWL	-	[78]
GGAGNDTLY	5, 9, 13, 17	[65]
